# Gendered Career Choices: Paths Toward Studying a Degree in Physical Activity and Sport Science

**DOI:** 10.3389/fpsyg.2019.01986

**Published:** 2019-09-11

**Authors:** Pedrona Serra, Susanna Soler, María José Camacho-Miñano, Ana Rey-Cao, Anna Vilanova

**Affiliations:** ^1^Institut Nacional d’Educació Física de Catalunya (INEFC), Universitat de Barcelona (UB), Barcelona, Spain; ^2^Department of Specific Didactics and Pedagogy, University of the Balearic Islands, Palma, Spain; ^3^Department for Languages, Arts and Physical Education, Feminist Research Institute, Complutense University of Madrid, Madrid, Spain; ^4^Department of Special Didactics, Universidade de Vigo, Pontevedra, Spain

**Keywords:** career choice, social support, gender differences, young people, physical education, interest

## Abstract

Drawing on Social Cognitive Career Theory (SCCT), we examined factors affecting interest in pursuing a degree in Physical Activity and Sport Science (PASS) among Spanish teenage students. Although women were awarded 55.1% of all bachelor degrees in Spain in 2017–2018, female enrollment in PASS degrees is decreasing and currently stands below 20% across the country. To better understand the under-representation of women in this field, 4146 students (50.2% girls; mean age = 16.82 years; *SD* = 0.837) participated in a survey designed to measure a series of SCCT constructs: interest in studying a PASS degree, career outcome expectations, goal representations, and perceived social supports. With these data, we tested a set of path analysis models to explain gender differences in interest in studying a degree in PASS. These models tested the assumption that interest in PASS would mediate the paths from outcome expectations and social supports to goal representations. Model 1 assumed that interest would partially mediate the path from outcome expectations to goal representations, Model 2 assumed complete mediation, and Model 3 assumed absence of mediation. All models were tested separately for boys and girls. Our results provide information on how male and female students set personal goals based on expected career outcomes and show that this process is affected by gender stereotypes. The lack of interest by young women in studying a degree in PASS (only 7.8% of girls expressed this interest compared with 19.0% of boys), together with the gender differences observed in perceived social supports, outcome expectations, and goal representations, have several important theoretical and practical implications. The present research suggests that interventions that foster positive outcome expectations and social support are necessary to increase interest in studying PASS among teenage girls.

## Introduction

Although women were awarded 55.09% of all bachelor’s degrees in Spain in 2017/18, they remain under-represented in science, technology, engineering, and mathematics (STEM) fields (Ministerio de Educación Cultura y Deporte, 2019). Female enrollment in physical activity and sport science (PASS) degrees in Spain is also worryingly low, at 18% (Serra et al., 2019). This tendency is even more alarming considering the fact that enrollment has been falling since 2000, when women accounted for 40% of all PASS students (Serra et al., 2019). This female under-representation is logically linked to a predominant male presence in sport professions additionally characterized by considerable horizontal and vertical gender segregation (Moragas, 2014; Viñas and Pérez, 2014; Pérez-Villalba et al., 2018). Women have also been found to be under-represented in coaching positions and similar professions in countries with different higher education and sports systems (Cunningham et al., 2005, 2007; Cunningham and Singer, 2010; Moran-Miller and Flores, 2011; Acosta and Carpenter, 2012; Hinojosa-Alcalde et al., 2017). A number of studies have addressed obstacles facing women interested in pursuing sporting careers, such as refereeing (Azurmendi, 2016; Kim and Hong, 2016), or occupying management and leadership positions in sporting organizations (Elling et al., 2019). This situation is not new, as in a study published over 10 years ago, Webb and Macdonald (2007) highlighted the difficulties faced by women in pursuing a successful career in physical education. In short, sport is largely characterized by gender biases, with a predominance of men and discrimination against women.

While Porto (2009) and Serra et al. (2019) have documented a downward trend in female enrollment in PASS degrees in recent years, no studies to date have explored the factors underlying the low presence of females in these degrees. To better understand the under-representation of women in this field, the aim of this study was to explore factors that influence male and female teenagers’ decisions to choose or not to choose to study a degree in PASS. The ultimate aim is to identify factors that need to be corrected in order to reduce the gender gap in PASS degrees and improve women’s representation in professional paths. Identification of these factors will guide corrective strategies aimed at improving female enrollment rates in this university degree and ultimately their presence in related professions.

## Theoretical Framework

Prior research has shown that social cognitive career theory (SCCT) (Lent et al., 1994) is a useful framework for investigating career choice processes. It has been widely applied in STEM research and is also used to investigate student intentions to enter the sports and leisure industry. As indicated by Alexander et al. (2011) and Cunningham et al. (2005, 2007), SCCT offers a social and heuristic framework for investigating academic and career choices, recognizes the social forces underpinning these choices, and helps to understand how social influences impact actions and motivations. Numerous studies have, furthermore, attempted to shed light on gender disparities in other disciplines, such as STEM (e.g., Su and Rounds, 2015; Park et al., 2018).

### General Social Cognitive Career Theory Framework

Social cognitive career theory emerged in 1994 (Lent et al., 1994) as a specification of Bandura (1986) general social cognitive theory to contribute to our understanding of how people develop academic and career interests from the perspective of three core tenets: self-efficacy, outcome expectations, and goal representations (Lent et al., 1994; Bandura, 1997; Lent, 2012; Lent and Brown, 2013).

Self-efficacy refers to a person’s beliefs about their ability to achieve a desired outcome (Bandura, 1987; Lent et al., 1994; Lent, 2012). These beliefs are closely linked to the nature of the actions required to achieve the outcome, the conditions required for these actions to be successful, the person’s perception of their abilities, and the efforts required to overcome setbacks (Bandura, 1987). Self-efficacy partly determines outcome expectations, as a person who believes he or she is capable of performing a task successfully is likely to expect a positive outcome.

Outcome expectations refer to what a person expects will happen if they act or behave in a specific way (Lent et al., 1994; Lent, 2012). They are related to the question “If I do this, what will happen?” These expectations can be physical (e.g., monetary rewards or job opportunities), social (e.g., social approval, status), or self-evaluative (e.g., self-satisfaction) (Bandura, 1987). According to Lent et al. (1994), self-evaluative expectations are the strongest determinants of actions a person will take toward the attainment of a goal (Lent et al., 1994). People’s academic or career choices tend to be governed by how they envisage the outcomes of their choices (Everhart and Chelladurai, 1998; Cunningham et al., 2005). Outcome expectations, in turn, help to establish personal goals (Lent et al., 1994).

Goal representations have an important role in self-regulating behavior, as they help a person to organize and guide their behavior in relation to the goals they have set. In brief, a person’s actions are not simply the result of automatic responses to given situations or stimuli. Lent et al. (1994) stressed that personal goals were always present, but were generally implicit. Goals drive motivation when perceived as attainable (Lent et al., 1994). Individuals pursuing a given academic or career path need goals in order to plan and direct their actions (Lent, 2012). How satisfied or dissatisfied they then feel with these actions will either strengthen their resolve or prompt them to revise their goals (Lent, 2012).

People develop interest in activities that they believe they can perform competently and that they expect will have a positive effect on their personal development and social status and esteem. As their interest grows, they will set goals accordingly. People who anticipate negative outcomes, however, or who do not believe themselves capable of achieving a given goal (Blanco, 2009), are unlikely to develop this interest. People, like their environments, are constantly changing, and therefore their actions and envisaged outcomes will influence their attitudes and opinions, and may even modify their environment (Alexander et al., 2011).

The three key determinants of academic interest development according to SCCT – self-efficacy, outcome expectations, and goal representations – are also strongly linked to social support received in relation to the pursuit of one path or another.

### Social Support in Relation to the Development of Academic and Career Interest

Social cognitive career theory posits that contextual factors have an important impact on academic and career choices (Lent et al., 1994). These factors may include family aspirations, financial situation, and level of education (Lent, 2012).

Social agents can also have a major influence on teenagers navigating academic and career choices (Stake and Nickens, 2005), in particular, family and teachers (Turner and Lapan, 2002; Sáinz, 2007; Metheny et al., 2008; Sáinz and López-Sáez, 2010; Rogers and Creed, 2011; Buday et al., 2012). Having friends with similar interests positively influences outcome expectations regarding certain professions (e.g., science professions) (Stake and Nickens, 2005), and social support from teachers, family, or friends can enhance a person’s self-efficacy beliefs, help them to set goals, and motivate them to pursue these goals (Buday et al., 2012).

Contextual factors also have an important effect on interest development. According to Holland (1997), people first tend to develop preferences for activities that are influenced by people they interact with in their social circles. These preferences gradually become interests as the person gains confidence in their ability to perform successfully (Nauta, 2013). Social supports can strengthen vocational interest and consequently drive motivation and goal pursuit, just as barriers can have the opposite effect (Cunningham et al., 2005).

All these contextual factors configure a strong gendered socialization process that mediate gender differences in vocational and career interests (Lent et al., 2001, 2010; Lent and Brown, 2013). According to Betz and Hackett (2006), young men and women differ in their vocational interests and goals because the opportunities presented to them from an early age also differ, and this affects self-efficacy beliefs. This theory would explain why boys and girls are generally less interested in careers that do not conform to traditional gender stereotypes (Lent, 2012).

### Previous Research in Women’s Career Development

Bandura (1986) self-efficacy theory was first applied to explain career choices in work by Betz and Hackett (2006) aimed at understanding career development in women. These authors hypothesized that career self-efficacy beliefs played a more powerful role than interests, values, or abilities in restricting women’s career choices. Girls exposed to traditional gender-role attitudes during childhood would have limited access to the information they need to develop strong self-efficacy beliefs in relation to a wide range of occupational fields. Gender differences in academic and career self-efficacy beliefs are linked to past gender-role socialization, current gender-role pressures, and perceptions of the gender-relatedness of tasks, activities, or occupations (Betz and Hackett, 1981). Traditional gender role attitudes and sterotypes regarding “appropriate” careers can undermine women’s beliefs that they can successfully pursue a non-traditional career. The stronger the perceived gender linkage of an activity or occupation, the more likely it is that gender differences in self-efficacy will arise (Bandura, 1997).

People are generally more likely to choose a particular academic or vocational path if they envision a favorable outcome (Cunningham et al., 2007). Girls and women often adopt “satisficing” strategies by choosing traditionally female occupations that are perceived to be easier to combine with home and family responsibilities rather than optimally translating their interests and abilities into career pursuits (Bandura, 1997). Furthermore, Betz and Hackett (2006) illustrated how the process of gender role socialization can bias access by both boys and girls to information they need to develop strong self-efficacy beliefs, particularly in relation to strongly gendered activities.

Research in the field of STEM has identified several factors that contribute to gender bias in academic and career choices (Morgan et al., 2001; Sáinz, 2007; Candela, 2008; Rogers et al., 2008; Eccles, 2011; Su and Rounds, 2015), with findings showing that girls largely choose a career based on personal preferences, while boys are more likely to be influenced by future prospects, such as earning a lot of money, doing or inventing new things, or becoming famous. Girls, by contrast, largely choose STEM careers where they can help others, reflecting the extent to which gender stereotypes influence career choice (Sallop and Kirby, 2007; Sáinz, 2007; Candela, 2008; López-Bonilla et al., 2012). Women thus are more likely to opt for social care or affiliated professions (Jones et al., 2000) and to gravitate away from traditionally male STEM professions (Sáinz, 2007).

The aim of the present study was to test a series of path models to detect factors that influence teenager boys’ and girls’ decisions to choose or not to choose to study a degree in PASS. In line with SCCT and our review of the literature, these models will be defined based on the following hypotheses: (H1) social supports will be significantly associated with interest; (H2) PASS outcome expectations will be positively related to interest; (H3) interest will partially mediate the paths from outcome expectations and social supports to goal representations; and (H4) gender differences will be observed for perceived social supports, outcome expectations, and goal representations. To test the last hypothesis, the path models were tested separately for boys and girls.

## Materials and Methods

### Sample and Data Collection

We analyzed a representative sample of students from 39 randomly selected secondary schools in three regions of Spain: Catalonia, Galicia, and Madrid (95% confidence interval; ±3). The schools were representative of a wide range of socioeconomic conditions (different social classes and urban and rural settings) and school types (public and private schools with and without vocational PASS courses).

All students enrolled at these schools in their final year of junior secondary education (4° *ESO*, 15–16 years old) and in the first of 2 years of the pre-university course (1° *Bachillerato*, 16–17 years old) were invited to participate. A total of 4146 students from 13 schools in each region took part in this study. The questionnaires were administered during class time by members of the research team. Girls accounted for 50.3% of the participants and no gender differences were observed in the distribution of the sample. Mean age was 16.82 years (*SD* = 0.837). Overall, 87.2% of participants were of Spanish origin; 3.37% were from other European countries (3.37%) while 9.40% were from countries in other parts of the world (e.g., Latin America and Africa). In total, 74.2% of the adolescents were from a school located in an urban area, while 28.5% were from a school in a rural area. Over two-thirds of the participants (67.7%) engaged in sport or exercise in their free time, but the rate was significantly higher for boys (76.3%) than girls (59.3%) [χ^2^(1) = 136.825, *p* < 0.0001]. The students had not participated in any careers guidance sessions before completing the questionnaire or received information about career opportunities in this field.

### Instruments and Measures

The instrument consisted of an *ad hoc* questionnaire designed to collect demographic information and data for measuring four variables in relation to studying a degree in PASS: (a) goal representations, (b) career outcome expectations, (c) perceived social support, and (d) interest in studying the degree.

The preliminary questionnaire was evaluated and validated by six experts in the field. Based on their feedback, it was then modified for the pilot test. The resulting questionnaire was piloted among 155 students from a school that did not participate in the study to ascertain length of completion and comprehensibility. It was then further refined for administration in the 39 schools. All results reported in this article are based on data collected in June 2014.

Goal representation and outcome expectations were measured using 15 items adapted from the scales developed by Everhart and Chelladurai (1998) and by Sáinz (2007), who translated Eccles and Harold (1991). Three of the variables were based on the outcome expectations described by Bandura (1986): social (e.g., to help other people), self-evaluative (e.g., to learn new things), and physical (e.g., to obtain power, monetary rewards, or status). Drawing on the work of Sáinz (2007), we added a fourth outcome expectation variable focused on job attributes that are specific to or characteristic of most jobs in the field of sport and exercise. The questions were worded in such a way that the students’ answers would provide information about their personal goals (e.g., “I would like a job that would allow me to…”) and their outcome expectations (e.g., “If I studied a degree in PASS, I think I would be able to get a job that allowed me to…”). Items were rated on a 5-point Likert-type scale that ranged from 1 (strongly disagree) to 5 (strongly agree).

Social supports perceived by the students in relation to their interest in studying a degree in PASS was assessed using two variables: one based on the work of Sáinz (2007) and another based on our review of the literature. The two questions were (1) Has anybody recommended that you study PASS when you finish school? and (2) Has anybody recommended that you don’t study PASS when you finish school? The options were “my father,” “my mother,” “my friends,” “my physical education teacher,” “another teacher,” “a coach,” and “other,” and the students had to answer “yes” or “no.”

Interest in studying a degree in PASS was assessed by a single item: Are you interested in studying a degree in PASS? The response options were: “yes,” “maybe,” “I don’t know yet,” and “no.” The first two options were grouped as indicating interest while the second two options were grouped as indicating a lack of interest.

### Data Analysis

We first performed a missing data analysis and a descriptive analysis of all study variables. Using the software package SPSS 18.0, we compared all variables between boys and girls using the *t-*test and assessed the internal consistency of the scales using Cronbach’s alpha.

Prior to the mediational analysis, we calculated the correlations between all the variables to be included in the path analysis models. Correlation coefficients were interpreted according to the criteria of Safrit and Wood (1995): no correlation (score of 0–0.19), low correlation (0.20–0.39), moderate correlation (0.40–0.59), moderately high correlation (0.60–0.79), and high correlation (≥0.80). We then tested a path analysis model in which the students’ interest in studying PASS was hypothesized to mediate the relationship (path) between outcome expectations and goal representations. In addition, we expected that perceived social support would act only as a predictor of interest. The correlations and path analysis models are presented separately for boys and girls.

According to MacKinnon (2008), a mediating variable “is intermediate in the causal path from an independent variable to a dependent variable” (p. 8). To test for mediation, we explored three *a priori* models: Model 1 to test for partial mediation (direct and indirect effects), Model 2 to test for complete mediation (indirect effects only, i.e., interest), and Model 3 to test for absence of mediation (direct effects only). All three models were tested using the maximum likelihood estimator. Goodness of fit to the data was assessed using the following model fit indices: χ^2^, root mean square error of approximation (RMSEA), the comparative fit index (CFI), and the Tucker-Lewis index (TLI). CFI and TLI values ≥0.95 and RMSEA values ≥0.06 were considered indicators of excellent fit; the respective values of ≥0.90 and ≤0.08 were considered to indicate acceptable fit.

Model 1 was the least parsimonious model and was therefore compared to Models 2 and 3. The comparisons were based on differences in χ^2^ and in CFI, TLI, and RMSEA values. The most parsimonious model was only selected when differences in CFI were <0.01 and when TLI or RMSEA values were as good as or better than those obtained for the least parsimonious model. Mediated effects were obtained using the Model Indirect command and the VIA instruction as defined in the Mplus 7.0 software (Muthén and Muthén, 2012).

## Results

### Missing Data, Descriptive Scale Statistics, and Internal Consistency

Missing data rates did not exceed 1.1% for any of the study variables and therefore, according to Graham (2009), will not have affected our data analyses because they were <5%. In subsequent analyses thus we used pairwise deletion of missing data. Table 1 shows the descriptive statistics for each study variable. Although the differences were small, they were all statistically significant except for self-evaluative goal representations. Of particular note were the differences in goal representations in the social (*M*_*boys*_ = 3.40; *M*_*girls*_ = 3.65), physical (*M*_*boys*_ = 3.72; *M*_*girls*_ = 3.62), and specific job attribute (*M*_*boys*_ = 2.97; *M*_*girls*_ = 2.53) domains. The difference for perceived social supports was also significantly different (*M*_*boys*_ = 0.83 and *M*_*girls*_ = 0.43). Table 2 shows Cronbach’s alphas for all the variables except the interest variable.

**TABLE 1:** Comparison between boys and girls for outcome expectations, perceived social supports, interest, and goal representations in relation to studying a degree in PASS.

	**Boys, *M* (*SD*)**	**Girls, *M* (*SD*)**	***t*-test: *t*(*df*), *p***
**Outcome expectations**			
1. Social	3.31 (0.58)	3.46 (0.56)	−8.456 (4079.778), <0.001
2. Self-evaluative	3.55 (0.83)	3.53 (0.84)	0.649 (4098), NS
3. Physical	3.23 (0.79)	3.10 (0.75)	5.342 (4076), <0.001
4. Specific job attributes	4.18 (0.76)	4.35 (0.80)	−6.880 (4102), <0.001
5. Perceived social supports	0.83 (1.43)	0.43 (1.06)	10.039 (3764.491), <0.001
6. Interest	1.65 (0.91)	1.31 (0.66)	13.811 (3720.206), <0.001
**Goal representations**			
7. Social	3.40 (0.72)	3.65 (0.71)	−10.846 (4055), <0.001
8. Self-evaluative	3.92 (0.74)	3.98 (0.69)	−2.611 (4046.626), 0.009
9. Physical	3.72 (0.75)	3.62 (0.75)	4.279 (4054), <0.001
10. Specific job attributes	2.97 (0.94)	2.53 (0.81)	15.723 (3966.968), <0.001

**TABLE 2:** Correlations between variables analyzed in relation studying a degree in PASS.

	**1**	**2**	**3**	**4**	**5**	**6**	**7**	**8**	**9**	**10**
**Outcome expectations**										
1. Social	*0.61*	0.388^∗∗^	0.215^∗∗^	0.541^∗∗^	0.105^∗∗^	0.124^∗∗^	0.338^∗∗^	0.294^∗∗^	0.187^∗∗^	0.145^∗∗^
2. Self-evaluative	0.424^∗∗^	*0.68*	0.572^∗∗^	0.213^∗∗^	0.183^∗∗^	0.221^∗∗^	0.371^∗∗^	0.371^∗∗^	0.207^∗∗^	0.324^∗∗^
3. Physical	0.312^∗∗^	0.526^∗∗^	*0.66*	0.057	0.184^∗∗^	0.246^∗∗^	0.360^∗∗^	0.256^∗∗^	0.310^∗∗^	0.398^∗∗^
4. Specific job attributes	0.537^∗∗^	0.129^∗∗^	–0.046	*0.73*	0.004	–0.007	0.134^∗∗^	0.197^∗∗^	0.158^∗∗^	0.100^∗∗^
5. Perceived social supports	0.008	0.138^∗∗^	0.158^∗∗^	–0.048	*0.79*	0.582	0.235^∗∗^	0.024	0.069^∗^	0.334^∗∗^
6. Interest^1^	0.013	0.162^∗∗^	0.176^∗∗^	–0.048	0.580^∗∗^		0.268^∗∗^	0.015	0.056	0.438^∗∗^
**Goal representations**										
7. Social	0.317^∗∗^	0.297^∗∗^	0.243^∗∗^	0.110^∗∗^	0.070^∗^	0.094^∗^	*0.63*	0.343^∗∗^	0.331^∗∗^	0.465^∗∗^
8. Self-evaluative	0.248^∗∗^	0.323^∗∗^	0.174^∗∗^	0.200^∗∗^	0.022	0.030	0.283^∗∗^	*0.59*	0.354^∗∗^	0.168^∗∗^
9. Physical	0.147^∗∗^	0.117^∗∗^	0.217^∗∗^	0.090^∗^	0.071^∗^	0.041	0.290^∗∗^	0.373^∗∗^	*0.68*	0.271^∗∗^
10. Specific job attributes	0.056	0.248^∗∗^	0.257^∗∗^	–0.026	0.330^∗∗^	0.415^∗∗^	0.288^∗∗^	0.210^∗∗^	0.148^∗∗^	*0.64*

*Correlations computed using data for girls are presented in the left/bottom triangle of the table. Correlations computed using data for boys are presented in the right/upper triangle of the table. The range for all variables is 1–5, except for social supports (1–6) and interest (1–4). Cronbach’s alpha coefficients are presented in the diagonal in italic. ^1^Interest was measured using a single item and therefore no Cronbach’s alpha coefficients are presented. *^∗^p* < 0.05, *^∗∗^p* < 0.01.*

### Correlations Between Variables

Almost all the correlations were positive and statistically significant (Table 2), although most of them were below <0.20 (no correlation) or between 0.20 and 0.39 (low correlation). Differences between male and female students were observed for correlations between outcome expectations and perceived social supports and interest.

### Mediational Models

For this study, we tested a series of mediated path models to study the predictors of interest among teenage boys and girls in studying a degree in PASS. Relationships between outcome expectations, social supports, interest, and goal representations were analyzed using three different path models. Model 1 assumed that the effect of outcome expectations on goal representations would be partially mediated by interest in studying a degree in PASS; Model 2 assumed complete mediation, while Model 3 assumed absence of mediation. This sequence of competing models was tested separately for boys and girls. The results depicting the fit of the models to the data are presented in Table 3.

**TABLE 3:** Model fit statistics and standardized coefficient estimates for mediation path analysis models.

**Model**	**χ^2^(*df*)**	**RMSEA (CI 90)**	**CFI**	**TLI**	**ΔRMSEA**	**ΔCFI**	**ΔTLI**
**Girls**							
1. Partial	16.455^∗^ (4)	0.055 (0.029–0.084)	0.990	0.914			
2. Complete	318.726^∗^ (20)	0.121 (0.110–0.133)	0.763	0.585	0.066	−0.227	−0.329
3. Direct	185.569^∗^ (12)	0.119 (0.105–0.135)	0.862	0.598	0.064	−0.128	−0.316
**Boys**							
1. Partial	12.567^∗^ (4)	0.047 (0.019–0.077)	0.995	0.954			
2. Complete	427.946^∗^ (20)	0.144 (0.132–0.156)	0.752	0.567	0.097	−0.243	−0.387
3. Direct	227.967^∗^ (12)	0.135 (0.120–0.151)	0.869	0.618	0.088	−0.126	0.377

*1. Partial = Model 1, partial mediation model; 2. Complete = Model 2, complete mediation model; 3. Direct = Model 3, direct effects model. *^∗^p* < 0.05.*

**FIGURE 1:**
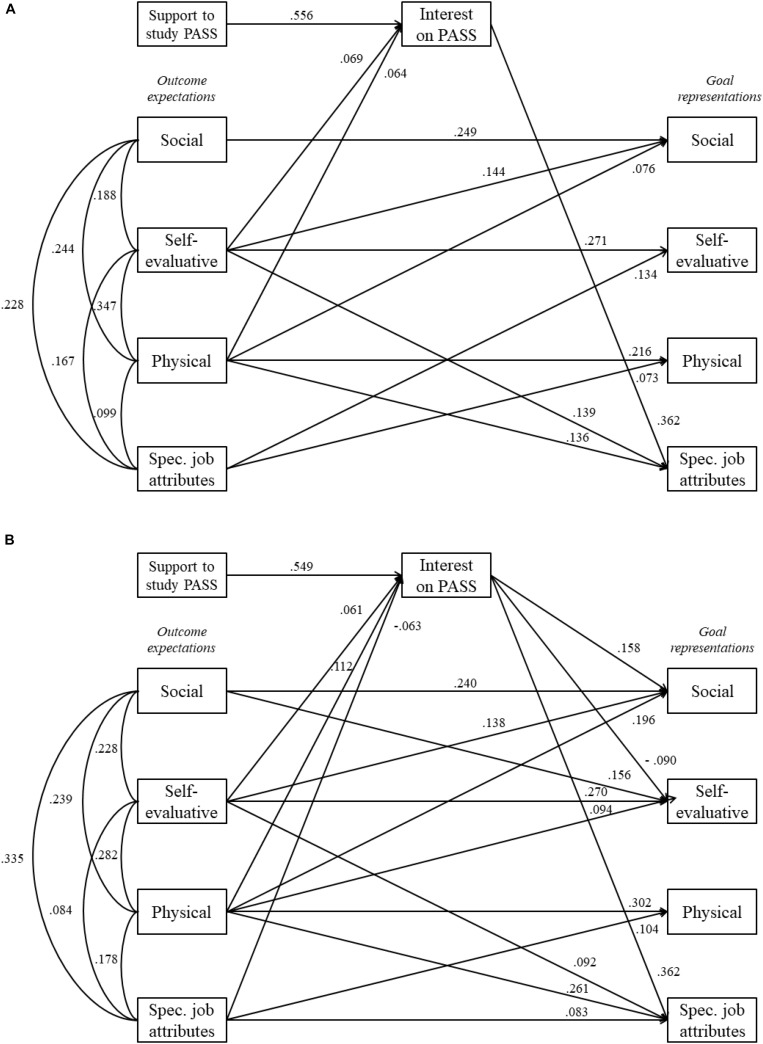
Path analysis models showing the partial mediation of girls’ **(A)** and boys’ **(B)** interest in studying a degree in physical activity and sport science in the paths from perceived social supports and outcome expectations to goal representations. Only standardized statistically significant paths (*p* < 0.05) are shown. Spec. job attributes, specific job attributes.

Model 1 provided an adequate fit for both boys and girls [boys: χ^2^ (*df*) = 12.567 (4), *p* < 0.001, RMSEA (90 CI) = 0.047 (0.019 –0.077), CFI = 0.995, TLI = 0.954; girls: χ^2^ (*df*) = 16.455 (4), *p* < 0.001, RMSEA (90 CI) = 0.055 (0.029 –0.084), CFI = 0.990, TLI = 0.914], and outperformed Models 2 and 3, supporting thus the hypothesis that interest in studying PASS partially mediated the effect of outcome expectations on goal representations (Figure 1). The *R*^2^ values for Model 1 were (a) boys – $R_{\text{social}\,\left(\mathrm{goal}\,\mathrm{representations}\right)}^{2}$ = 0.240, $R_{\mathrm{self}-\mathrm{evaluative}\,\left(\mathrm{goal}\,\mathrm{representations}\right)}^{2}$ = 0.181, $R_{\text{physical}\,\left(\mathrm{goal}\,\mathrm{representations}\right)}^{2}$ = 0.119, $R_{\mathrm{specific}\,\mathrm{job}\,\mathrm{attributes}\,\left(\mathrm{goal}\,\mathrm{representations}\right)}^{2}$ = 0.297, and $R_{\text{interest}}^{2}$ = 0.370; and (b) girls – $R_{\mathrm{social}\,\mathrm{supports}\,\left(\mathrm{goal}\,\mathrm{representations}\right)}^{2}$ = 0.138, $R_{\mathrm{self}-\mathrm{evaluative}\,\left(\mathrm{goal}\,\mathrm{representations}\right)}^{2}$ = 0.127, $R_{\text{physical}\,\left(\mathrm{goal}\,\mathrm{representations}\right)}^{2}$ = 0.057, $R_{\mathrm{specific}\,\mathrm{job}\,\mathrm{attributes}\,\left(\mathrm{goal}\,\mathrm{representations}\right)}^{2}$ = 0.215, and $R_{\text{interest}}^{2}$ = 0.344. Therefore, the boys’ model explained slightly more variance than the girls’ model.

The statistically significant effects observed in Model 1 (Figures 1A,B and Table 4) showed some differences between girls and boys. The boys’ model contained more direct and indirect (i.e., mediated by interest) effects. The following effects, all significant, were observed in the boys’ model only: (a) indirect effect of perceived support on social (0.087) and self-evaluative (−0.049) goal representations; (b) direct effect of social outcome expectations on self-evaluative goal representations (β = 0.156) and indirect effect on specific job attributes (0.022); (c) direct (β = 0.094) and indirect (−0.010) effect of physical outcome expectations on self-evaluative goal representations and indirect effect on social goal representations (0.018); (d) direct effect of specific job attribute outcome expectations on interest (β = −0.063) and specific job attribute goal representations (β = 0.083) and indirect effect on social (−0.010) and specific job attribute (−0.023) goal representations. In the girls’ model, self-evaluative outcome expectations had an indirect effect on specific job attribute goal representations (0.025), while specific job attribute outcome expectations had a direct effect on self-evaluative goal representations (β = −0.134).

**TABLE 4:** Standardized indirect effects of outcome expectations on goal representations.

**Independent variable**	**Mediator variable**	**Dependent variable**	**Boys**		**Girls**	
				
			**Mean indirect effect**	**95% CI for mean indirect effect**	**Mean indirect effect**	**95% CI for mean indirect effect**
Perceived social supports	Interest	Social GRs	0.087^∗^	0.053–0.120	0.026	−0.006–0.058
Perceived social supports	Interest	Self-evaluative GRs	−0.049^∗^	−0.082 to −0.017	−0.003	−0.036–0.030
Perceived social supports	Interest	Physical GRs	−0.014	−0.044–0.016	0.005	−0.023–0.034
Perceived social supports	Interest	Specific job attribute GRs	0.199^∗^	0.161–0.236	0.201^∗^	0.149–0.253
Social OEs	Interest	Social GRs	0.010	−0.001–0.020	−0.001	−0.005–0.002
Social OEs	Interest	Self-evaluative GRs	−0.006	−0.012–0.001	0.000	−0.003–0.003
Social OEs	Interest	Physical GRs	−0.002	−0.006–0.002	0.000	−0.002–0.002
Social OEs	Interest	Specific job attribute GRs	0.022^∗^	0.001–0.043	−0.010	−0.033–0.012
Self-evaluative OEs	Interest	Social GRs	0.008	−0.002–0.018	0.003	−0.002–0.008
Self-evaluative OEs	Interest	Self-evaluative GRs	−0.005	−0.011–0.002	0.000	−0.005–0.004
Self-evaluative OEs	Interest	Physical GRs	−0.001	−0.005–0.002	0.001	−0.003–0.005
Self-evaluative OEs	Interest	Specific job attribute GRs	0.019	−0.004–0.041	0.025^∗^	0.005–0.045
Physical OEs	Interest	Social GRs	0.018^∗^	0.005–0.030	0.003	−0.002–0.008
Physical OEs	Interest	Self-evaluative GRs	−0.010^∗^	−0.018 to −0.002	0.000	−0.005–0.004
Physical OEs	Interest	Physical GRs	−0.003	−0.009–0.004	0.001	−0.003–0.004
Physical OEs	Interest	Specific job attribute GRs	0.040^∗^	0.017–0.064	0.023^∗^	0.001–0.045
Specific job attribute OEs	Interest	Social GRs	−0.010^∗^	−0.020–0.000	−0.001	−0.005–0.003
Specific job attribute OEs	Interest	Self-evaluative GRs	0.006	−0.001–0.012	0.000	−0.002–0.002
Specific job attribute OEs	Interest	Physical GRs	0.002	−0.002–0.006	0.000	−0.002–0.002
Specific job attribute OEs	Interest	Specific job attribute GRs	−0.023^∗^	−0.043 to −0.003	−0.007	−0.031–0.017

*Perceived social support, perceived supports in relation to studying a degree in sport and exercise science; Interest, interest in studying a degree in sport and exercise science; OEs, outcome expectations; GRs, goal representations. *^∗^p* < 0.05.*

Some common paths were also observed for boys and girls in the form of the following effects: (a) direct effect of perceived social supports on interest (β_*girls*_ = 0.556, β_*boys*_ = 0.549) and indirect effect on specific job attributes (β_*girls*_ = 0.201; β_*boys*_ = 0.199); (b) direct effect of social outcome expectations on social goal representations (β_*girls*_ = 0.249, β_*boys*_ = 0.240); (c) direct effect of self-evaluative outcome expectations on interest (β_*girls*_ = 0.069, β_*boys*_ = 0.061) and on social (β_*girls*_ = 0.144, β_*boys*_ = 0.138), self-evaluative (β_*girls*_ = 0.271, β_*boys*_ = 0.270), and specific job attribute (β_*girls*_ = 0.139, β_*boys*_ = 0.092) goal representations; (d) direct effect of physical outcome expectations on interest (β_*girls*_ = 0.064, β_*boys*_ = 0.112) and social (β_*girls*_ = 0.076, β_*boys*_ = 0.196), physical (β_*girls*_ = 0.216, β_*boys*_ = 0.302), and specific job attribute (β_*girls*_ = 0.136, β_*boys*_ = 261) goal representations and indirect effect on specific job attribute goal representations (β_*girls*_ = 0.023, β_*boys*_ = 0.040); and (e) direct effect of specific job attribute outcome expectations on physical goal representations (β_*girls*_ = 0.073, β_*boys*_ = 0.104).

## Discussion

The findings of this study contribute to our understanding of why female teenagers are less interested in studying a degree in PASS than their male peers and have important theoretical and practical implications.

Perceived social support was an important predictor of interest in studying PASS in both the boys and girls analyzed. In agreement with the literature, the empirical model showed that perceived support was significantly associated with interest, confirming thus our first hypothesis (H1). As indicated by Lent et al. (2000), social context has an important role in the development of interest toward one academic track or another. Social support, however, differs for boys and girls. Therefore, the potential influence of significant others (e.g., teachers, trainers, families, and friends) on a teenagers’ academic and career choices should not be overlooked (Turner and Lapan, 2002; Stake and Nickens, 2005; Sáinz, 2007; Metheny et al., 2008; Sáinz and López-Sáez, 2010; Rogers and Creed, 2011; Buday et al., 2012).

Social cognitive career theory (Lent et al., 1994) posits that outcome expectations predict academic and career interests and goal representations. Our findings show that these expectations were significantly associated with goal representations both directly and indirectly (with interest as a mediator), confirming thus our second and third hypotheses (H2 and H3). We also observed gender differences in specific outcome expectation and goal representation variables. Self-evaluative and physical outcome expectations were significant in the paths identified for both girls and boys, but the boys’ model additionally featured expectations related to specific job attributes. SCCT also posits that outcome expectations will exert a direct effect on goal representations in relation to career choices (Lent et al., 2001; Lent, 2012). In our study, we observed gender differences for each of the variables in the model, and as expected, the specific job attribute outcome expectation was related to personal goal representations among boys only.

The above findings provide insights into how teenage boys and girls establish personal goals based on the outcomes they envisage in relation to studying a degree in PASS. It has been well established that academic and career choices among both girls and boys are influenced by gender stereotypes. As observed in the literature review, female students are more likely to choose people-oriented careers, while male students are generally more interested in wealth and competitive positions (Sallop and Kirby, 2007; Sáinz, 2007; Candela, 2008; López-Bonilla et al., 2012). Our results also indicate that male and female teenagers are influenced by stereotyped portrayals of PASS studies and associated careers. Even though work in this field is largely people-oriented, it does not appear to be perceived as such by young people. Consequently, and in agreement with findings for STEM (Sáinz et al., 2014), misconceptions may discourage females from pursuing a career in this field.

Our results also support the theory that interest influences goal representations. SCCT predicts that interest in a given academic track or career will directly affect the setting of personal goals. In our study, goal representations were influenced by both interest and outcome expectations, confirming our third hypothesis (H3). However, again, the results for girls and boys differed. In the girls’ model, interest in pursuing a degree in PASS was associated with job-specific attribute expectations only, while in the boys’ model, it was associated with social, self-evaluative, and specific job attribute expectations.

Our fourth hypothesis (H4) was also confirmed, as we detected gender differences in perceived social supports, outcome expectations, and goal representations. We also found that the boy’s model had greater explanatory power in relation to the SCCT than the girls’. While the girls’ model provided an adequate and significant fit to the data, its paths were less elaborate than the boys’. Overall, and in support of work by Betz and Hackett (2006), our study confirms the importance of applying gender analysis to all the components of SCCT and its respective models.

Our findings support the applicability of SCCT to PASS studies and provide new insights into associated career choices among teenage boys and girls in their final years of school. They also support claims by Betz and Hackett (1981) that gender stereotypes influence perceptions of tasks, activities, and occupations, and extend current theories on social support. Social agents are known to exert a powerful influence on academic and career choices (Turner and Lapan, 2002; Sáinz, 2007; Sáinz and López-Sáez, 2010; Buday et al., 2012; Lent, 2012).

The results of our study confirm that male and female teenagers perceive different levels of support from their social environment in relation to their intention to study PASS at university. As expected, our path analysis showed gender differences along the path from social support to interest in these studies. Consistent with findings for research in the field of STEM, the effect of perceived social supports on the development of interest in studying PASS also differed between males and females (Sáinz and López-Sáez, 2010), with girls receiving less encouragement from people in their social circles to contemplate a career in PASS.

### Practical Contributions

The findings of this study are relevant to educational institutions and career counselors seeking to bridge the gender gap in the field of PASS. PASS graduates can opt for a wide range of jobs in the fields of health, education, and care delivery, and while these professions have traditionally attracted women, female enrollment in PASS degrees has steadily declined over the past 25 years (Serra et al., 2019). Strategies targeting social agents are needed to break down gender-role stereotypes and encourage more young women to contemplate a career in this field. Institutions seeking to attract more women thus need to move away from the male-centered sports culture typically transmitted to society. Awareness-raising programs highlighting the myriad of job opportunities associated with PASS could also improve outcome expectations among young women.

People already working in this field, whether in academia or industry, can also play an important role in transforming overly simplistic and gendered conceptions of PASS. By serving as role models, they can foster positive outcome expectations and inspire young women to consider pursuing a career in this field. Training of these future professionals, however, needs to integrate a gender perspective, as otherwise, they may become agents of reproduction rather than change. The inclusion of gender issues in PASS studies and training programs for physical education instructors is crucial for breaking down gender stereotypes and widening the perspectives of future trainers. Numerous other factors in the teenagers’ social and school environment, including positive experiences with sport and physical activity, can also help teenagers to develop an interest in studying PASS. If this dichotomous, male-centered vision of physical education, sport, and exercise continues, these degree courses will continue to be dominated by males.

Our findings are also relevant for sports policymakers and employers as sport and exercise permeate all levels of society; offer multiple job opportunities; and are of enormous economic, social, and cultural importance. If women are not encouraged to enroll in PASS and similar degrees, their participation in the workforce will decrease even further, resulting in a loss of richness and diversity that will only perpetuate existing gender gaps. Diversity drives innovation and creativity, and applied to the field of PASS, it may also provide inspiration to young women and other people from varying sociocultural backgrounds. A key practical implication of our study is that our findings can provide institutions and practitioners with insights into how best they can tailor initiatives to attract more women to PASS degrees (Serra et al., 2018; Soler et al., 2018).

Combating the under-representation of females in PASS degrees remains a challenge that, if correctly addressed, will contribute to eliminating gendered academic and career choices among young women and men.

### Limitations and Future Directions

There are several limitations that should be noted in this study. First of all, we measured interest as an intention not a behavior. It would therefore be interesting to perform a longitudinal study of the adolescents who participated in this study to monitor their academic and career paths and see how they progress toward the attainment of their goals. It would also be interesting to perform a similar study from the perspective of the SCCT performance model, which analyzes persistence in educational and occupational pursuits and level of success achieved (performance outcomes, satisfaction, and well-being) (Lent et al., 1994). Analysis of these additional factors would help better understand the career choice process from a wider perspective. It would be particularly interesting to analyze how people who set themselves specific academic or career goals persist in the pursuit of these goals, regardless of positive or negative social influences or of perceived self-efficacy in physical activity and sport. Detailed comparisons of strategies followed by male and female adolescents could shed light on how and why the few girls interested in studying a degree in PASS actually go on to study this degree, despite perceived physical and social barriers. In relation to the academic/career satisfaction/well-being SCCT model (Lent and Brown, 2006; Lent, 2012), it would be interesting to analyze similarities and differences among male and female PASS students in terms of (1) value placed on the activity chosen, (2) awareness of progress toward personal goals, (3) beliefs in their ability to perform well in necessary tasks (self-efficacy), and (4) access to resources in their environment that enhance their self-efficacy beliefs and drive them to pursue their goals with greater energy (Lent and Brown, 2006).

Based on our findings, we suggest numerous areas for future research. Of particular interest is further investigation of external factors (social supports) that directly influence the development of interest in studying PASS among girls and boys. A closer analysis of the degree of influence exerted by different social agents (parents, siblings, trainers, teachers, etc.) will provide important insights into the relevance of each agent. Qualitative research based on interviews or women-only focus groups may also shed light on perceived social supports and barriers that encourage or discourage teenage girls from pursuing a career in PASS.

To conclude, the results of this study support many of the hypothesized relationships between the SCCT variables analyzed and suggest that both perceived social supports and positive conceptions of outcome expectations are important predictors of the formulation of personal goals in relation to studying a degree in PASS. Our findings contribute to a broader understanding of the SCCT model and show that while the basic relationships are generally confirmed, the different elements have a particular influence on social supports (environmental/external factors) and outcome expectations.

We have explored factors that influence male and female teenagers’ interest in pursuing a degree in PASS. Trends from recent years indicate that if the current situation continues, female enrollment in these degrees will continue to decline and with it the number of potential role models for future generations.

By better understanding how teenage girls develop interest in PASS, we will be better equipped to create strategies that will attract women to this field and in doing so contribute to bridging the gender gap.

## Data Availability

The datasets generated for this study are available on request to the corresponding author.

## Ethics Statement

The study was reviewed and approved by the Research Ethics Committee of the Sports Administration of Catalonia, and ethical concerns were addressed throughout the research process. Written informed consent to participate in this study was provided by the participants’ legal guardian/next of kin. Respondents and their families were provided with information about the study and were assured of confidentiality of identifiable information and were informed of their right to withdraw participation at any time without prejudice from any party. Informed signed consent was obtained from the students’ parents and the school authorities prior to the study. No incentives were offered.

## Author Contributions

PS, SS, MC-M, AR-C, and AV: design of the study, analysis and interpretation of the data, and manuscript preparation. PS: acquisition of the data. SS: conceptualization and obtaining funding.

## Conflict of Interest Statement

The authors declare that the research was conducted in the absence of any commercial or financial relationships that could be construed as a potential conflict of interest. The handling Editor declared a shared affiliation, though no other collaboration, with one of the authors, PS, at the time of the review.
